# Human cardiac telocytes: 3D imaging by FIB-SEM tomography

**DOI:** 10.1111/jcmm.12468

**Published:** 2014-10-17

**Authors:** D Cretoiu, E Hummel, H Zimmermann, M Gherghiceanu, L M Popescu

**Affiliations:** a Department of Cell Biology and Histology, Carol Davila University of Medicine and PharmacyBucharest, Romania; b ‘Victor Babeş’ National Institute of PathologyBucharest, Romania; c Carl Zeiss Microscopy GmbHMunich, Germany

**Keywords:** telocytes, heart, myocardium, FIB-SEM tomography, 3D imaging

## Abstract

Telocyte (TC) is a newly identified type of cell in the cardiac interstitium (www.telocytes.com). TCs are described by classical transmission electron microscopy as cells with very thin and long telopodes (Tps; cellular prolongations) having podoms (dilations) and podomers (very thin segments). TCs’ three-dimensional (3D) morphology is still unknown. Cardiac TCs seem to be particularly involved in long and short distance intercellular signalling and, therefore, their 3D architecture is important for understanding their spatial connections. Using focused ion beam scanning electron microscopy (FIB-SEM) we show, for the first time, the whole ultrastructural anatomy of cardiac TCs. 3D reconstruction of cardiac TCs by FIB-SEM tomography confirms that they have long, narrow but flattened (ribbon-like) telopodes, with humps generated by the podoms. FIB-SEM tomography also confirms the network made by TCs in the cardiac interstitium through adherens junctions. This study provides the first FIB-SEM tomography of a human cell type.

## Introduction

Telocytes (TCs) are a novel type of interstitial cells described by transmission electron microscopy (TEM) in heart 1–8 and many other organs of vertebrates 9–18 (see www.telocytes.com).

The shortest definition of TCs is cells with telopodes (Tps). These Tps are extremely long prolongations (several tens to hundreds of micrometers) with podomers (ultrathin segments below the resolving power of light microscopy) and podoms (dilated portions containing mitochondria and endoplasmic reticulum). An up-to-date review is available 19.

During the last few years, focused ion beam scanning electron microscopy (FIB-SEM) became the election technique for 3D visualization of biological structures at nanoscale resolution 20,21. FIB-SEM tomography is the most promising approach for 3D imaging at the subcellular level and is considered as a true revolution for ultrastructural volume reconstruction 22. Briefly, FIB-SEM setups offer a series of successive ultrastructural images using concomitantly a focused ion beam for slicing and an electron beam for imaging.

Anyway, FIB-SEM tomography seems the ‘ideal’ available method to disclose the arborescent conformation of TCs (describes on 2D ultrathin sections).

## Material and methods

### Sample preparation

Small human heart samples (atrial appendages) were obtained from the patients undergoing heart surgery for congenital heart diseases. The small samples of myocardium were processed as previously described 5. Briefly, the 1-mm-cube fragments were fixed by immersion in 4% glutaraldehyde, and post-fixed in 1% OsO_4_ with 1.5% K_4_Fe(CN)_6_ (potassium ferrocyanide – reduced osmium) to increase the membranes contrast. Subsequently, the samples were dehydrated through increasing graded ethanol series and embedded in epoxy resin (Agar 100 from Agar Scientific, Essex, UK) at 60°C for 48 hrs.

### FIB/SEM image stack acquisition

Focused ion beam milling and SEM imaging were carried out with a ZEISS Auriga Crossbeam system (from Carl Zeiss Microscopy, München, Germany). FIB milling was performed with 600 pA to 20 nA for the given samples. SEM-Imaging current was 220 pA. To achieve the best signal contrast, the mixed Inlens and energy-selective backscattered detector signals were used. FIB milling steps was 10 nm/slice and each 5th slice was imaged. Accordingly, each image represents 50 nm of the stack, at 9kX magnification. Image pixel size was 10.27 nm.

### Stack alignment, segmentation and 3D presentation

Images were first sorted into stacks according to sections alignments for re-alignment. Then, images were processed using Adobe Photoshop CS6 (Adobe Systems Incorporated, San Jose, CA, USA) for re-alignment, noise detection and removal, luminance level adjustment and cropping by regions of interest. Images prepared were then loaded by batches into 3D Slicer 4.3.1 (64 bit; Harvard Medical School, Boston, MA, USA) 23 software package (http://www.slicer.org) and reconstructed using Volume Rendering module 24. Parameters of the Volume Rendering module were set according to the luminance level of the structures of interest (cells), leaving the background (intercellular space) transparent. Stacks of images were also loaded in VirtualDub v1.10.4 (Lee A.) software 25 as sequence of numbered JPEG files and converted to video file.

## Results and discussion

Previous TEM studies showed that telopodes, cellular prolongations of TC, are very long, predominantly narrow (usually about 100 nm) and accommodate mitochondria and endoplasmic reticulum in small dilations named podoms (usually less than 1 μm width). Often the Tps were observed to be discontinuous in 2D images obtained from 60 nm thin serial section by TEM and this suggested a tubular aspect of Tp. Serial sectioning and 3D reconstruction using TEM is not a practical solution to solve the 3D architecture as a result of the dimension of cell (up to 100 μm).

**Figure 1:**
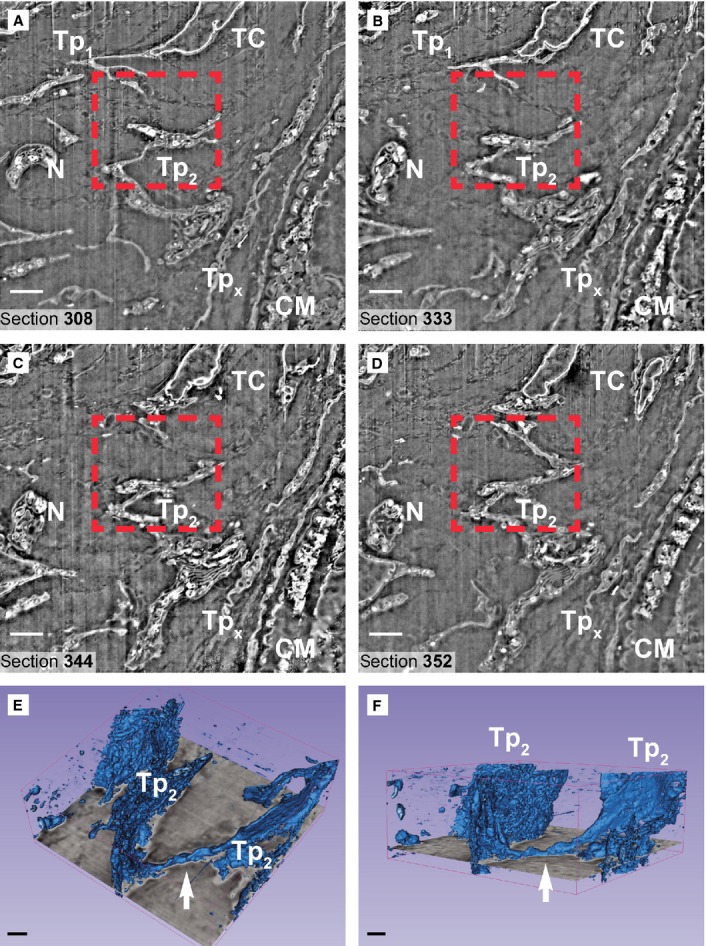
(**A**–**D**) Four non-consecutive serial images obtained in backscattered electron imaging mode show a telocyte (TC) with two telopodes (Tp1, Tp2). The telopode Tp2 is about 30 μm long and has a waving trajectory. The Tp2 connection with the cell body of the TC is not visible on digital slices (**A**–**C**). The FIB-SEM tomography (rectangular mark in **A**–**D**) and 3D reconstruction (arrows in **E** and **F**) show the link between apparent disconnected segments of Tp2 in 2D analysis (transmission electron microscopy). Tpx – a telopode which belongs to a different TC, N-nerve ending, CM – cardiomyocyte. Scale bars: 2 μm.

**Figure 2:**
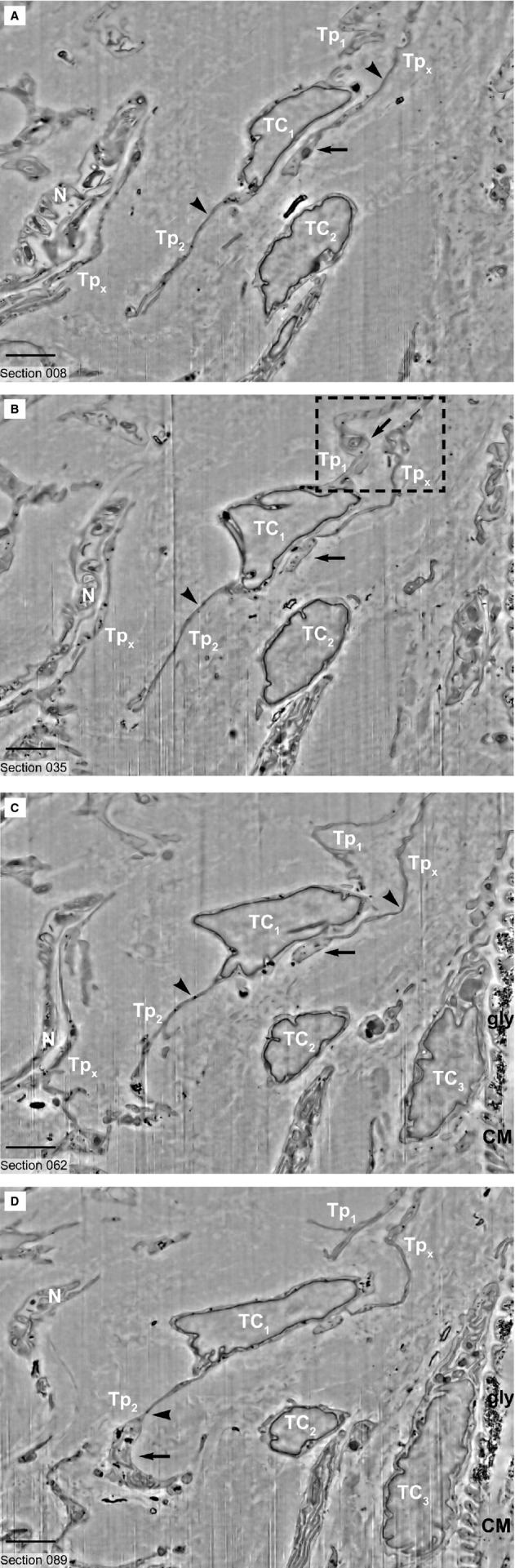
(**A**–**D**) FIB-SEM images (inverted) show 3 telocytes (TC1, TC2, TC3) and a nerve ending (N) in the vicinity of a cardiomyocyte (CM). In 2D digital slices extracted from the volume, the telocyte TC1 show typical narrow segments (podomers, arrowheads) of the telopodes (Tp1, Tp2) alternating with dilations (podom, arrows). The narrow emergence of telopode Tp1 from TC1 is visible in **B**. The telocyte TC1 is reconstructed in Figure 3. Rectangular marked area is reconstructed in Figure 4. Tpx – telopodes belonging to different telocytes, N-nerve ending, CM – cardiomyocyte, gly- glycogen particles in CM. Scale bars: 2 μm.

For this reason, FIB-SEM tomography, a technology which allows volume investigation, was carried out with a ZEISS Auriga Crossbeam system on plastic embedded human cardiac tissue. An area surrounding a blood vessel has been selected for investigation, since TCs are preferentially located in the perivascular region. The cardiac tissue investigated had the following dimensions: x – 20.77 μm; y – 21.01 μm; z – 25 μm; area – 436.38 μm^2^; volume – 10,908.57 μm^3^. The backscattered electron imaging mode at 50 nm z-interval generated a stack of 500 serial images with 10.27 nm resolution at 9.000 magnification step. The FIB-SEM images showed numerous cells with long and thin prolongations, with typical morphology for TCs, present in the perivascular space of atrial tissue (Figs 1 and 2; Video S1).

**Figure 3:**
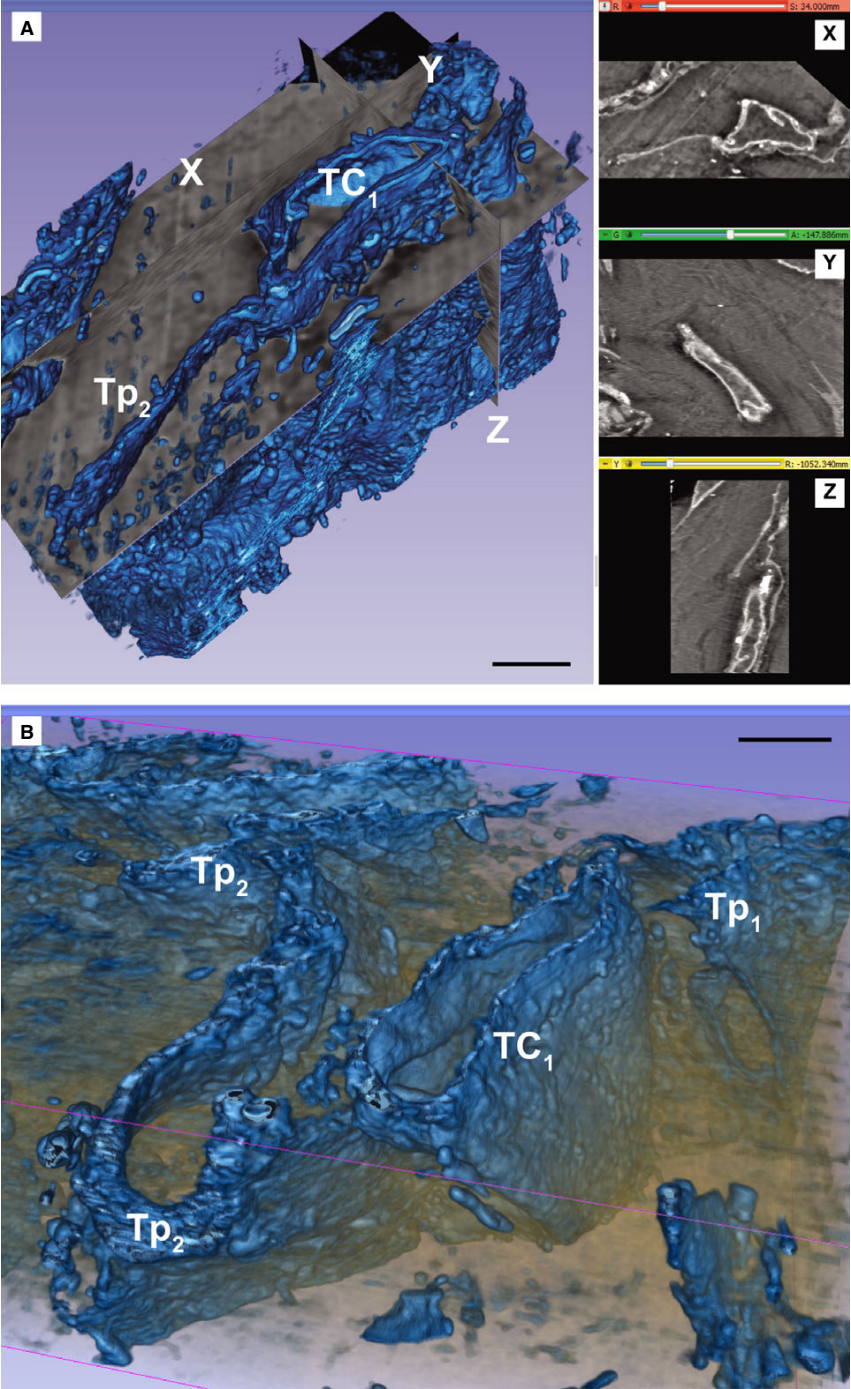
(**A** and **B**) Automated segmentation of the stack containing the telocyte TC1 from Figure 2 shows that the telopode Tp2 is long (20 μm), narrow (0.2–1 μm) and flat, given a ribbon appearance of the cell. X-Y-Z slice projections from volume could be seen in the right side of **A**. Scale bars: 2 μm.

**Figure 4:**
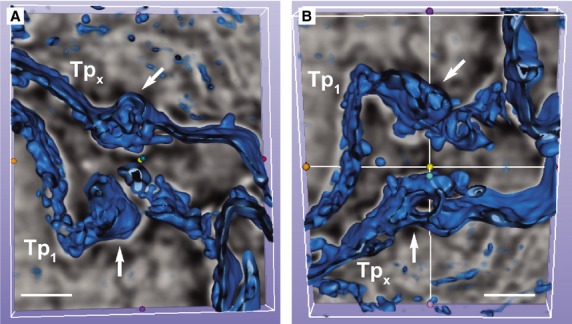
(**A** and **B**) Automated segmentation of the stack containing telopodes (Tp1, Tpx) from rectangle marked in Figure 2 shows how podoms prominence disrupt the flatness of the telopodes. Scale bar: 1 μm.

**Figure 5:**
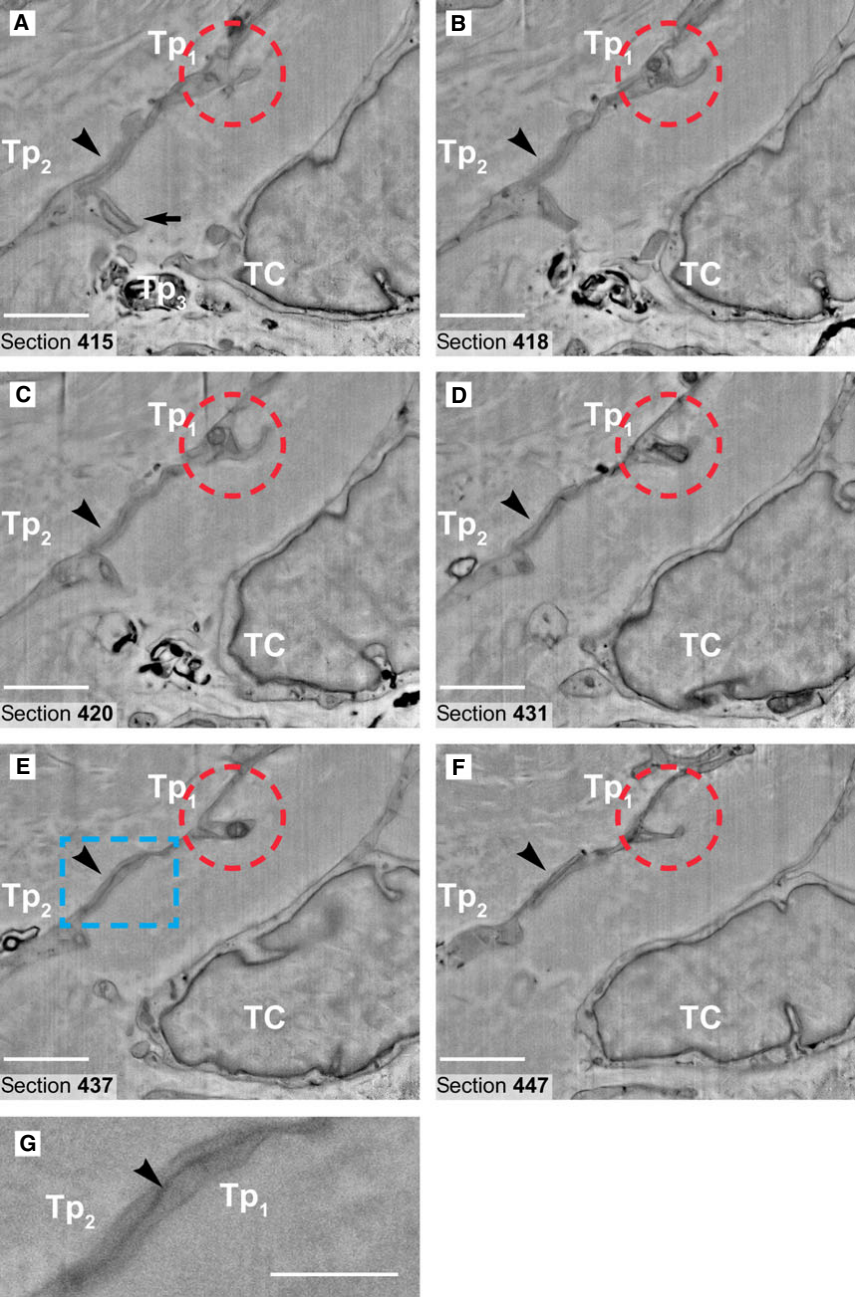
(**A**–**F**) Six non-consecutive serial images (inverted) obtained in backscattered electron imaging mode at 150 nm z-interval. The quality of the images in FIB-SEM is comparable with classical transmission electron micrograph at 9kX magnification. The red ring indicates a characteristic dilation (podom) of the telopode Tp1, where intracellular structures such as endoplasmic reticulum cisternae and mitochondria are visible. A junction (processus adherens type) could be seen connecting the telopodes Tp1 and Tp2 (arrowheads). The area of this junction (rectangular marked area in **E** is enlarged in **G**) is about 5 μm2 (2 μm/2.5 μm). Another emerging junction (recessus adherens type) is visible (arrow in **A**) between telopodes Tp2 and Tp3 of the adjoining telocyte (TC). Scale bars: **A**–**F**, 2 μm; **G**, 1 μm.

FIB-SEM tomography showed that TCs have narrow and flat cellular prolongations (Video S1). 3D reconstruction of a cardiac TC (Fig. 3) showed that Tps have mostly a ribbon-like conformation and that podoms bulge from the podomer plane (Figs 4 and 5). FIB-SEM also confirms previous data showing the presence of mitochondria and endoplasmic reticulum within podoms 5,15,26,27. The analysis of serial sections showed that TCs are connected each other (Fig. 5) and form a 3D network (Video S1) as reported previously 5,6,28. Moreover, we have found that intercellular connections between TCs usually occur through wide adherens junctions (Fig. 5) most likely to increase the stability of the network. Also, the close vicinity with nerve endings 5,28 was found.

Last but not least, this study provides clear evidence that TCs are completely different from fibroblasts, as reported previously, as concerns microRNA imprint 29, gene profile 30–32 and proteomics 33. The dynamics of Tps in cell culture is dissimilar for TCs compared to fibroblasts prolongations 34.

In conclusion, the dual-beam FIB-SEM instrumentation, associated with increased computer power and sophisticated display options, appears at present as a quintessential tool for the shift from 2D ultrathin sections to 3D analysis of ultrastructural volumes. Thus, the complex conformation of TCs (Tps, podoms and ribbon-like podomers) and their labyrinthine 3D network is revealed.

## Supporting Information

Video S1 FIB-SEM of the telocyte TC1 from Fig. 2 and reconstructed in Fig. 3. 150 slice (50nm) sequence of realigned, inverted micrographs with enhanced contrast. Round marks in the movie point out to the connections between the telopodes of telocytes from Fig. 2.
